# Quantitative Correlations between Radiosensitivity Biomarkers Show That the ATM Protein Kinase Is Strongly Involved in the Radiotoxicities Observed after Radiotherapy

**DOI:** 10.3390/ijms231810434

**Published:** 2022-09-09

**Authors:** Eymeric Le Reun, Larry Bodgi, Adeline Granzotto, Laurène Sonzogni, Mélanie L. Ferlazzo, Joëlle Al-Choboq, Laura El-Nachef, Juliette Restier-Verlet, Elise Berthel, Clément Devic, Audrey Bouchet, Michel Bourguignon, Nicolas Foray

**Affiliations:** 1 Inserm, U1296 Unit, «Radiation: Defense, Health and Environment», Centre Léon-Bérard, 28, rue Laennec, 69008 Lyon, France; 2 Department of Radiation Oncology, American University of Beirut Medical Center, Riad El-Solh, Beirut 1107-2020, Lebanon; 3 Department of Anatomy, Cell Biology and Physiological Sciences, Faculty of Medicine, American University of Beirut, Bliss Street, 11-0236 Riad El-Solh, Beirut 1107-2020, Lebanon; 4 Department of Biophysics and Nuclear Medicine, Université Paris Saclay Versailles St. Quentin en Yvelines, 78035 Versailles, France

**Keywords:** radiosensitivity, radiotherapy, DNA double-strand breaks, ATM, overreactions, predictive assays

## Abstract

Tissue overreactions (OR), whether called adverse effects, radiotoxicity, or radiosensitivity reactions, may occur during or after anti-cancer radiotherapy (RT). They represent a medical, economic, and societal issue and raise the question of individual response to radiation. To predict and prevent them are among the major tasks of radiobiologists. To this aim, radiobiologists have developed a number of predictive assays involving different cellular models and endpoints. To date, while no consensus has been reached to consider one assay as the best predictor of the OR occurrence and severity, radiation oncologists have proposed consensual scales to quantify OR in six different grades of severity, whatever the organ/tissue concerned and their early/late features. This is notably the case with the Common Terminology Criteria for Adverse Events (CTCAE). Few radiobiological studies have used the CTCAE scale as a clinical endpoint to evaluate the statistical robustness of the molecular and cellular predictive assays in the largest range of human radiosensitivity. Here, by using 200 untransformed skin fibroblast cell lines derived from RT-treated cancer patients eliciting OR in the six CTCAE grades range, correlations between CTCAE grades and the major molecular and cellular endpoints proposed to predict OR (namely, cell survival at 2 Gy (SF2), yields of micronuclei, recognized and unrepaired DSBs assessed by immunofluorescence with γH2AX and pATM markers) were examined. To our knowledge, this was the first time that the major radiosensitivity endpoints were compared together with the same cohort and irradiation conditions. Both SF2 and the maximal number of pATM foci reached after 2 Gy appear to be the best predictors of the OR, whatever the CTCAE grades range. All these major radiosensitivity endpoints are mathematically linked in a single mechanistic model of individual response to radiation in which the ATM kinase plays a major role.

## 1. Introduction

Among cancer patients treated with radiation therapy (RT), about 5 to 20% may elicit tissue overreactions (OR) (also called adverse effects, radiotoxicity, or radiosensitivity reactions) occurring during or after the treatment. Such ORs can limit the application of the scheduled treatment and increase morbidity: ORs represent therefore a significant medical, economic, and societal issue [1,2,3,4,5,6,7,8,9,10]. One of the major tasks of radiobiologists is to better understand, predict, and prevent them [11]. However, the diversity of the predictive assays proposed, the number of different experimental protocols, cohorts, and cellular models, the biases linked to the extrapolation of data from animal models, and the lack of a systematic biomathematical approach that would justify and consolidate each step of the prediction process have not helped radiobiologists to reach a consensus about the predictive assay to be applied in routine [3,11,12].

First, the existence of ORs, which is likely to be specific to each individual, has long been debated: since ORs may be similar to the tissue reactions expected after a dose excess, ORs have been suggested to be caused by dosimetry errors. However, the RT accident in Epinal (France) demonstrated that the same dose excess may produce a large spectrum of OR severities among treated patients, likely reflecting individual responses to RT [13,14]. This last statement must be modulated by the considerable progress in the quantification of the physical dose of ionizing radiation (IR) in the irradiated tissue area. In addition, a number of genetic diseases associated with significant radiosensitivity have highlighted the impact of individual predisposition in the final outcome of RT-treated patients, independently of any dosimetry error [3,15].

In order to establish the requirements for a relevant and robust prediction of ORs, let us survey the major features of the post-RT ORs based on clinical observations:

- *Spectrum of OR severities*: ORs show a large and continuous spectrum of severities, from non-hazardous effects on irradiated healthy tissues to fatal reactions. Hence, any relevant predictive assay should reflect this spectrum with constant statistical robustness. In other terms, the power of OR prediction of the predictive assays should be independent of the OR severity [3].
- *Dose–response*: The higher the dose, the more severe and the earlier the OR. Such observations suggest that predictive assays should also be consistent with the dose–response relationships observed both in vivo and in vitro [5].
- *Early/late OR prediction*: The early or late occurrence of ORs does not condition their severities. In other terms, both early and late ORs may show a large spectrum of severities, from non-hazardous effects on irradiated healthy tissues to fatal reactions. Hence, the power of the OR prediction of the predictive assays should be independent of the early or late nature of ORs [6,16,17].
- *Tissue representativeness*: ORs potentially concern all the irradiated organs/tissues of the body. Hence, the molecular and cellular models chosen for the predictive assay should be representative enough to account for ORs occurring in all the tissues of the human body [3].
- *OR severity scales endpoint*: In practice, OR occurrence and severity are generally estimated with the local experience of each oncologist and each medical staff of each radiotherapy department. To alleviate the subjectivity of such approaches, some grading severity scales have been proposed to characterize post-RT ORs for each irradiated organ/tissue of the body. This is notably the case of the Common Terminology Criteria for Adverse Events (CTCAE) [16,17] and the Radiation Therapy Oncology Group (RTOG) [6] scales, which are the most extensively used. These two scales consist of classifying OR severity into six grades (from grade 0: no event, to grade 5: death). Hence, to establish a statistically robust correlation between the clinical features of ORs and molecular and/or cellular assays, the OR severity scales should be systematically used as clinical endpoints to quantify the individual response of each treated patient to RT [6,16,17].

Several approaches have been chosen by radiobiologists to develop predictive assays [3,11]. Among them, the *genomic* approach based on the determination of DNA sequences whether through single nucleotide polymorphisms (SNPs) or genome-wide association studies (GWAS) cannot reach the above dose–response requirement since DNA sequences, whatever their form, cannot predict the dose–response specific to each tissue and individual since it does not change after irradiation [18,19]. Hence, one SNP cannot be associated with a unique dose–response of a given healthy tissue for a given RT schedule. Conversely, a genomic approach consisting of a systematic analysis of all the mutations of all the genes potentially involved in radiotoxicity is interesting, particularly if some mutations are found in common with patients with the same nature and occurrence of OR [3]. However, even if the genotype/phenotype relationships are well known for one specific gene, such an approach would not reach the above dose–response requirement because many other mutated genes can interplay to provide a specific dose–response observed in each patient. Lastly, a genomic approach that would consist of quantifying the dose-dependent expression of one given gene involved in the individual response to IR is very promising, but further investigations are still needed to better understand the interplay with the radiation-induced expression of other genes involved in the final outcome [20,21].

Unlike the genomic approach, the *functional* approach privileges the assessment of biological functions essential for a normal individual response to IR, *independently of the knowledge of the DNA sequence* or the genes involved [3]. Such a “blind” approach reaches all the above requirements but does not directly provide a diagnosis of the mutations of the gene(s) that cause(s) ORs. The data accumulated all along the history of radiobiology converge to propose the clonogenic cell survival assay as the gold standard for predicting individual response to IR. The clonogenic cell survival assay is considered as belonging to the functional approach since it is based on the assessment of the whole radiation-induced (RI) cell death, independently of its nature (mitotic death, senescence, or apoptosis) [22,23,24,25,26,27]. Unfortunately, the clonogenic cell survival assay is too time-consuming to ensure a routine prediction of ORs. The same conclusions were reached with predictive assays based on cytogenetics endpoints, similarly to the micronuclei and the chromosome breaks assays that have been also found correlated to cell survival [3,28,29]. Lastly, since cell survival, micronuclei, and chromosome breaks were found to be correlated with unrepaired DNA double-strand breaks (DSBs), the yield of unrepaired DSBs may be of interest to predicting ORs [30,31,32,33,34,35]. However, according to the technique applied, there are numerous DSB endpoints proposed in the literature [36,37,38,39,40]. This is notably the case of immunofluorescence against the phosphorylated forms of the variant X of the H2AX histone (γH2AX) [38,41] and the phosphorylated forms of the ATM protein (pATM) [42] that both form nuclear foci at the DSB sites. Unfortunately, low yields of unrepaired DSBs were not accurate in predicting moderate radiosensitivity [40,43].

ATM kinase, whose mutations cause ataxia telangiectasia, the human syndrome associated with the highest radiosensitivity, is upstream of the major DSB repair and signaling pathways [44,45,46]. Since 2003, our group has accumulated hundreds of skin fibroblasts from RT-treated patients eliciting a large range of OR severities, the COPERNIC collection [35]. From the COPERNIC collection, we have provided experimental and theoretical clues showing that the radiation-induced (RI) nucleoshuttling of ATM (RIANS) is a statistically robust and reliable predictor of OR [43,47,48]. Lastly, it is noteworthy that the RIANS model is also at the basis of a novel resolution of the linear quadratic (LQ) model, the general formula that aims to link cell survival and radiation dose and whose biological interpretation has remained unsolved since the 1970s [49,50].

Here, with 200 COPERNIC skin fibroblast cell lines deriving from OR patients, inter-correlations between CTCAE grades, cell survival, micronuclei, and yields of recognized and unrepaired DSBs from γH2AX and pATM data were examined. A general, coherent, and multi-parametric analysis of the prediction of post-RT OR is proposed from quantitative correlations between the above radiosensitivity endpoints.

## 2. Results

### 2.1. Clonogenic Cell Survival vs. CTCAE Grades

**Figure 1:**
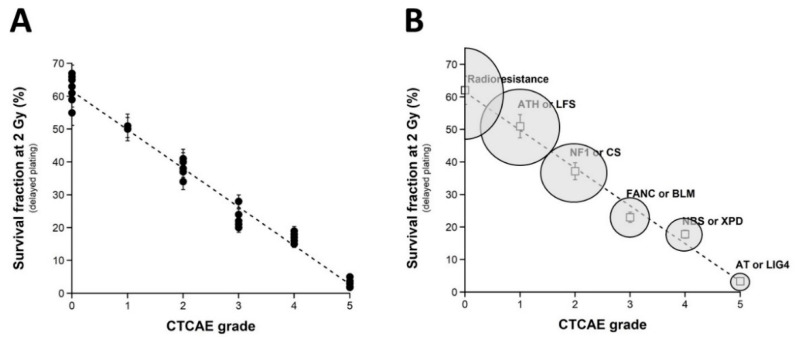
Clonogenic cell survival vs. CTCAE grades. (**A**) The SF2 data from 36 COPERNIC cell lines were plotted against the corresponding CTCAE grade values (closed circles). Each point corresponds to the mean ± standard error of the mean (SEM) of three independent triplicates, at least. The best data fit was obtained with the linear law: SF2 (%) = 61.55 − 11.72 × grade; r^2^ = 0.98 (dotted line). (**B**) The average SF2 values (±SEM) of the data shown in Fig1A were plotted against the corresponding CTCAE grade (open squares). The dotted line is the reproduction of the data fit shown in (**A**). An arbitrary 25% relative error was applied to the SF2 and grade values. The resulting 25% confidence circle zones were built in grey. They schematically reproduce the average SF2 values observed from fibroblasts deriving from patients suffering from the indicated syndromes (AT: ataxia telangiectasia, homozygous mutations of *ATM*; LIG4: homozygous mutations of *LIG4*; NBS: Nijmegen’s syndrome, homozygous mutations of *NBS1*; XPD, xeroderma pigmentosum D, homozygous mutations of *XPD*; BLM, Bloom’s syndrome, homozygous mutations of *BLM*; FANC, Fanconi anemia, homozygous mutations of *FANC*; NF1, neurofibromatosis type 1, heterozygous mutations of neurofibromin; CS, Cockayne’s syndrome, homozygous mutations of *CS*; LFS: Li-Fraumeni’s syndrome, heterozygous mutations of p53; ATH, heterozygous mutations of *ATM*. The syndromes data were obtained from our lab and published elsewhere [3,15].

Since a dose of 2 Gy X-rays generally represents the current dose applied per standard radiotherapy session, the survival fraction at 2 Gy (SF2) has been used to quantify radiosensitivity [22,24]. Here, the clonogenic cell survival assay was not systematically applied to all the cell lines of the COPERNIC collection but to a representative subset of 36 COPERNIC cell lines (including 10, 1, 6, 6, 6, and 7 cell lines derived from patients with CTCAE grade 0, 1, 2, 3, and 6 reactions, respectively (Figure 1A)). It must be stressed that, in practice, the clinical criteria of CTCAE grade 1 still remain subjective and some grade 1 ORs may be preferentially and provisionally graded to 0 or 2 CTCAE grade. No significant correlation was observed between the clonogenicity (plating efficiency) and the corresponding CTCAE grades, suggesting that the proliferation capacity is not a predictor of OR. The SF2 data were plotted against the corresponding CTCAE grades. The SF2 data from the patients who were apparently healthy or did not show ORs were not found significantly different (*p* > 0.8), and the corresponding SF2 values were in agreement with the most radioresistant cells in the literature (average SF2 value: 62.1 ± 1.4%; CTCAE grade 0). The SF2 values corresponding to patients who succumbed to RT (here, *ATM*- and *LIG4*-mutated patients) were also found consistent with published data (average SF2 value: 3.3 ± 0.5%; CTCAE grade 5). Hence, to obey the mathematical constraints, these bounded values suggested either a linear or a sigmoidal law between SF2 data and CTCAE grades. By plotting all the SF2 data available, the best data fit chosen among the current mathematical laws was found to be a linear law (Table 1):

SF2 (%) = 61.55 − 11.72 × grade; r^2^ = 0.98. (1)

Whatever the variation of grade 1 data, a sigmoidal law between SF2 and CTCAE grades was not possible. Altogether, our data suggest that decreasing every 11.72% cell survival increment leads to a decrease in one additional CTCAE grade (Figure 1A). It must be stressed here that such a conclusion was reached in the largest OR severity range possible. Altogether, these data consolidate the power of the OR prediction of the clonogenic cell survival assay. Interestingly, by calculating the average SF2 corresponding to each grade data (grade 0: 62.1 ± 1.4%; grade 1: 51.0%; grade 2: 37.2 ± 3.8%; grade 3: 23.0 ± 1.4%; grade 4: 17.8 ± 0.6%), some similarities appeared with the SF2 values associated with some well-characterized genetic diseases and the database of our research group [15]. These diseases are mentioned in Figure 1B. Such findings strengthened the fact that ORs and the radiosensitivity of genetic origin reflect the same large and continuous spectrum of responses to IR. Reciprocally, the CTCAE grades showed the relevant values that can be found in the case of the IR exposure of the patients suffering from the indicated genetic diseases associated with radiosensitivity (Figure 1B).

It is noteworthy that no fibroblast cell line of the COPERNIC collection elicited apoptotic death, whether irradiated or not. Since the COPERNIC fibroblasts cover the largest OR severity range possible, the absence of apoptosis suggests that this specific cellular death cannot explain radiotoxicity observed in fibroblasts, which represents the majority of human tissues.

### 2.2. Number of Micronuclei vs. CTCAE Grades and SF2-Micronuclei Relationships

**Figure 2:**
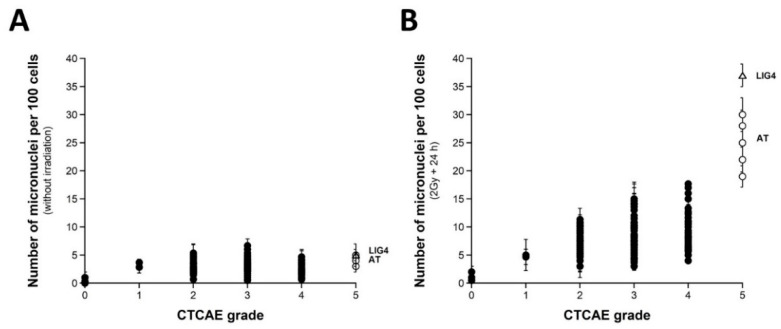
Micronuclei vs. CTCAE grades. The number of spontaneous micronuclei per 100 cells (**A**) or micronuclei assessed 24 h post-irradiation per 100 cells (**B**) from 200 COPERNIC cell lines were plotted against the corresponding CTCAE grade values (closed circles). Each point corresponds to the mean ± standard error of the mean (SEM) of three independent triplicates, at least. The points corresponding to the *ATM*- (open circles) and the *LIG4*- (open triangles) mutated cell lines are indicated.

Micronuclei lead to irreversibly damaged chromosomal fragments causing mitotic death [51]. Micronuclei have been shown to be quantitatively correlated with cellular radiosensitivity when assessed by the clonogenic cell survival assay [28,52,53,54]. However, to our knowledge, such cytogenetic endpoints have not yet been tested in a large spectrum of CTCAE grades, with the notable exception of our previous report gathering 117 COPERNIC cell lines [43]. Here, micronuclei were scored before or 24 h after irradiation in 200 COPERNIC cells. With regard to spontaneous micronuclei, no significant correlation was found with the CTCAE grades (Figure 2A). Conversely, when 24 h data were plotted against the CTCAE grades, a tendency appeared suggesting that the higher the number of micronuclei assessed at 24 h post-irradiation, the larger the CTCAE grade. However, the prediction of the CTCAE grades from the residual micronuclei was not reliable, with the notable exception of grades 0 and 5 ORs (Figure 2B). In addition, Table 2 showed that the number of micronuclei assessed 24 h post-irradiation cannot discriminate grade 2 from grade 3 and grade 3 from grade 4 ORs at a high degree of significance. The mathematical law that would link the number of micronuclei assessed at 24 h post-irradiation to CTCAE grades appeared complex. Further analysis will be discussed in the next chapters.

**Figure 3:**
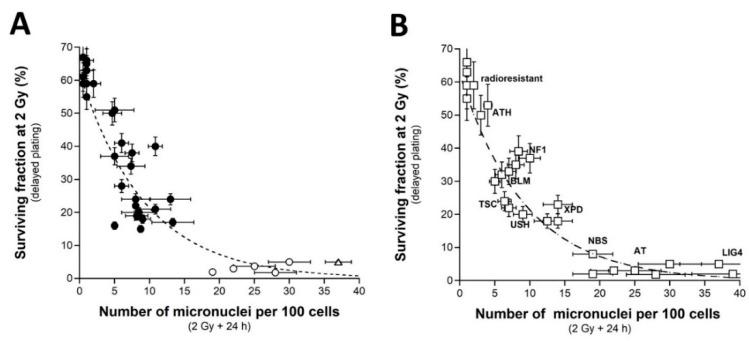
SF2 vs. micronuclei. (**A**) The SF2 data from 36 COPERNIC cell lines were plotted against the corresponding number of micronuclei per 100 cells assessed 24 h post-irradiation (closed circles). Each point corresponds to the mean ± standard error of the mean (SEM) of three independent triplicates, at least. The best data fit was obtained with the linear law: SF2 (%) = 62.2 exp (−0.107 MN_24h_); r^2^ = 0.939 (dotted line). The points corresponding to the *ATM*- (open circles) and the *LIG4*- (open triangles) mutated cell lines are indicated. (**B**) The SF2 values (±SEM) and the corresponding numbers of micronuclei per 100 cells assessed 24 h post-irradiation (±SEM) from fibroblasts deriving from patients suffering from the indicated syndromes (open squares) (AT: ataxia telangiectasia, homozygous mutations of *ATM*; LIG4: homozygous mutations of *LIG4*; NBS: Nijmegen’s syndrome, homozygous mutations of *NBS1*; XPD, xeroderma pigmentosum D, homozygous mutations of *XPD*; USH, Usher’s syndrome, homozygous mutations of USH; TSC, tuberous sclerosis, heterozygous mutation of *TSC*; Bloom’s syndrome, homozygous mutations of *BLM*; NF1, neurofibromatosis type 1, heterozygous mutations of neurofibromin; ATH, heterozygous mutations of *ATM*. The dotted line is the reproduction of the data fit shown in (**A**). The syndromes data were obtained from our lab and published elsewhere [3,15,30,43].

Since a correlation between SF2 and micronuclei has been previously established, we investigated the link between these two endpoints by plotting SF2 data against micronuclei assessed at 24 h post-irradiation. Among the current mathematical laws, an exponential law appeared to provide the best fit to link SF2 and the number of micronuclei assessed 24 h post-irradiation (MN_24h_) (Figure 3A) (Table 1):

SF2 (%) = 62.2 exp (−0.107 × MN_24h_); r^2^ = 0.939 (2)

Such a mathematical law is relevant whatever the CTCAE grade and appeared also relevant for fitting data from cell lines provided from genetic syndromes associated with radiosensitivity (published data, [3,15,30,43]), supporting again the fact that radiosensitivity from genetic syndromes obeys the same mathematical laws as radiotoxicity observed in cells from RT-treated patients (Figure 3B).

### 2.3. Number of H2AX Foci vs. CTCAE Grades and SF2-Micronuclei-γH2AX Foci Relationships

**Figure 4:**
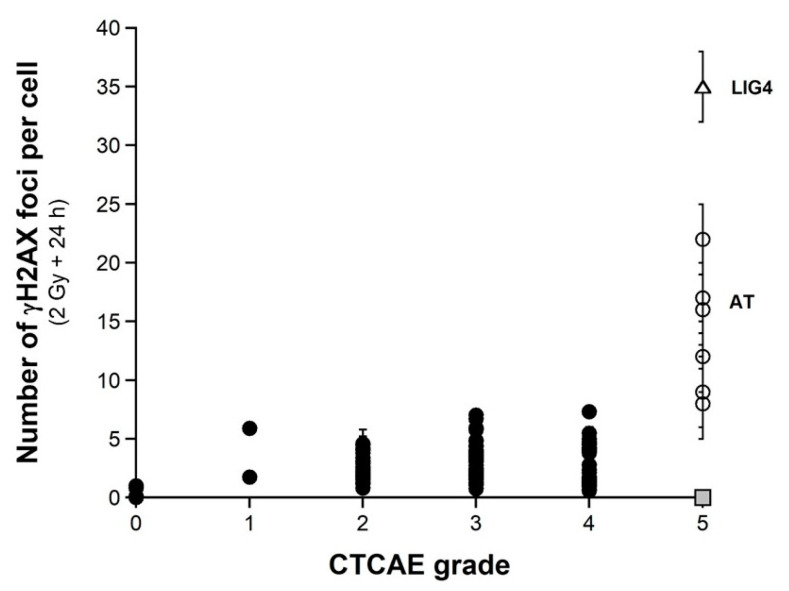
γH2AX foci vs. CTCAE grades. The number of γH2AX foci assessed 24 h post-irradiation from 200 COPERNIC cell lines were plotted against the corresponding CTCAE grade values (closed circles). Each point corresponds to the mean ± standard error of the mean (SEM) of three independent triplicates, at least. The points corresponding to the *ATM*- (open circles) and the *LIG4*- (open triangles) mutated cell lines are indicated. The grey square indicates all the AT data if considering γH2AX foci in *ATM*-mutated cells as absent.

As evoked above, one of the most current ways of assessing DSBs is the immunofluorescence assay performed with the anti-γH2AX antibody [38,41,55,56,57,58]. However, while IR “physically” induces about 40 DSBs per Gy in all the human fibroblasts, whatever their radiosensitivity, the number of γH2AX foci assessed early (10 min) after irradiation may depend on their radiosensitivity [43]. The nuclear γH2AX foci have been associated with the DSBs recognized by the ATM-dependent non-homologous end-joining (NHEJ), the most predominant DSB repair and signaling pathways in human quiescent cells [38,41,55,56,57,58]. In human radioresistant quiescent fibroblast cell lines, about 40 nuclear γH2AX foci per Gy per cell have been currently scored early after irradiation. This value was found similar to those deduced from other techniques assessing DSBs, which strongly supports that the anti-γH2AX immunofluorescence permits to detect the great majority of RI DSBs as far as the NHEJ pathway is predominant in the cells tested [3]. However, in radiosensitive cells, the amount of DSBs recognized by NHEJ was shown to be lower than the number of DSBs “physically” induced by IR [42,43], suggesting an impairment in the DSB recognition by NHEJ in these cells [42,43]. We, therefore, examined the number of early and residual γH2AX foci in the 200 COPERNIC cells. The COPERNIC cells have been exposed to 2 Gy X-rays followed by 10 min, 1, 4 (not shown), and 24 h for repair. No significant correlation was found between spontaneous γH2AX foci and CTCAE grades, in agreement with previous data [43]. The number of γH2AX foci assessed 24 h post-irradiation (H2AX_24h_) was plotted against the corresponding CTCAE grade (Figure 4). In agreement with the literature, the radioresistant controls showed the lowest H2AX_24h_ [41]. Cells from *ATM*-mutated patients did not show γH2AX foci (since the phosphorylation of H2AX is strongly ATM-dependent) or, if any, γH2AX foci were found very tiny and dispersed [43,59]. Hence, the number of γH2AX foci in *ATM*-mutated cells can be considered either as nil (grey square in Figure 4) by defining the γH2AX foci as similar to that of the other cell lines or as non-nil by taking into account the tiny γH2AX foci. In the fibroblast cell line (180BR) derived from a *LIG4*-mutated patient who succumbed to RT, the γH2AX foci data were found to be very specific: while the 180BR cells showed an SF2 value similar to those of the *ATM*-mutated cell lines, they elicited a number of early γH2AX foci similar to that observed in radioresistant controls (which suggests a normal DSB recognition) but with a number of residual γH2AX foci higher than 30 (which suggests a gross DSB repair defect). These data were found in agreement with literature that has provided a relevant interpretation of these specific radiobiological features: in fact, in 180BR cells, the recognition step is not affected by the *LIG4* mutations while the DSB repair step is strongly impaired [60,61,62]. All the other COPERNIC cell lines that correspond to grades 1–4 showed intermediate H2AX_24h_ [43]. As for micronuclei, there was a tendency of H2AX_24h_ to increase with the CTCAE grade. However, unlike with micronuclei, statistical analysis showed that H2AX_24h_ can discriminate between grades 2 and 3, and 3 and 4 but not between grades 2 and 4 (Table 2). Similarly for micronuclei, the mathematical law that would link γH2AX_24h_ with CTCAE grades appeared complex. Further mathematical analysis will be discussed in the next chapters.

**Figure 5:**
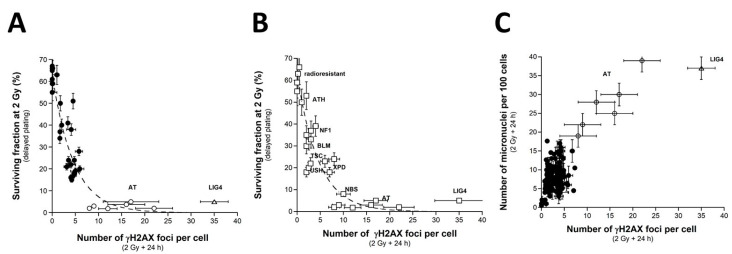
SF2 vs. γH2AX foci and micronuclei vs. γH2AX foci (**A**) The SF2 data from 36 COPERNIC cell lines were plotted against the corresponding number of γH2AX foci per cell assessed 24 h post-irradiation (closed circles). Each point corresponds to the mean ± standard error of the mean (SEM) of three independent triplicates, at least. The best data fit was obtained with the linear law: SF2 (%) = 62.56 exp (−0.216 H2AX_24h_); r^2^ = 0.87 (dotted line). (**B**) The SF2 values (±SEM) and the γH2AX foci assessed 24 h post-irradiation (±SEM) from fibroblasts deriving from patients suffering from the indicated syndromes (open squares) (AT: ataxia telangiectasia, homozygous mutations of *ATM*; LIG4: homozygous mutations of *LIG4*; NBS: Nijmegen’s syndrome, homozygous mutations of *NBS1*; XPD, xeroderma pigmentosum D, homozygous mutations of *XPD*; USH, Usher’s syndrome, homozygous mutations of USH; TSC, tuberous sclerosis, heterozygous mutation of *TSC*; Bloom’s syndrome, homozygous mutations of *BLM*; NF1, neurofibromatosis type 1, heterozygous mutations of neurofibromin; ATH, heterozygous mutations of *ATM*). The dotted line is the reproduction of the data fit shown in (**A**). The syndromes data were obtained from our lab and published elsewhere [3,15,30,43]. (**C**) The number of micronuclei assessed 24 h post-irradiation shown in Figure 2A (from 200 COPERNIC cell lines) was plotted against the corresponding numbers of γH2AX foci per cell assessed 24 h post-irradiation shown in Figure 4. The points corresponding to the *ATM*- (open circles) and the *LIG4*- (open triangles) mutated cell lines are indicated.

Since a correlation between SF2 and unrepaired DSBs has been established already [30], we investigated the link between these two endpoints by plotting SF2 data against γH2AX_24h_. Among the current mathematical laws, an exponential function appeared to provide the best fit between SF2 and γH2AX_24h_ (Figure 5A) (Table 1):

SF2 (%) = 62.56 exp (−0.216 × γH2AX_24h_); r^2^ = 0.87 (3)

Such mathematical law did not change with the pattern of the γH2AX foci defined from the *ATM*-mutated cells. These data strongly suggested that the SF2 decreased with γH2AX_24h_. However, for the cases showing intermediate radiosensitivity, while the corresponding SF2 values ranged from 7 to 60%, γH2AX_24h_ varied only from 0 to 10%. In other terms, the intermediate radiosensitivity is poorly predicted by γH2AX_24h_ but provides higher statistical performance than the micronuclei assay (Table 2). This mathematical law was found to be relevant to fit data from fibroblasts deriving from genetic syndromes (Figure 5B). Again, these findings support that radiotoxicity observed after RT and radiosensitivity observed in cells deriving from genetic syndromes obey similar quantitative links between cellular and molecular endpoints of radiosensitivity.

When plotted against γH2AX_24h_, MN_24h_ obeyed a linear or else a curvilinear function depending on whether the data corresponding to the *LIG4*-mutated cell line has been taken into account. Further mathematical analysis will be discussed in the next chapters. Such correlation shows that the γH2AX foci and/or else micronuclei are not relevant to predict the cases corresponding to the OR with CTCAE grades ranging from 1 to 4 (Figure 5C).

### 2.4. Number of pATM Foci vs. CTCAE Grades and SF2-Micronuclei-γH2AX Foci vs. pATM Foci Relationships

**Figure 6:**
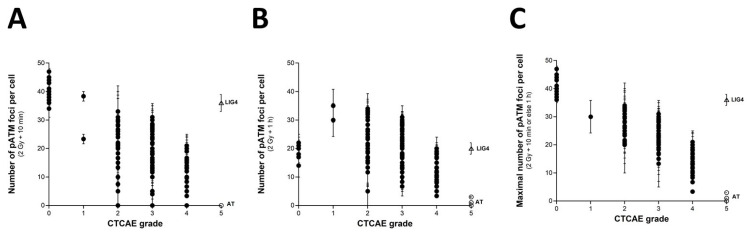
γH2AX foci vs. CTCAE grades. The number of pATM foci per cell assessed 10 min (**A**) or 1 h (**B**) post-irradiation from 200 COPERNIC cell lines was plotted against the corresponding CTCAE grade values (closed circles). Each point corresponds to the mean ± standard error of the mean (SEM) of 3 independent triplicates, at least. (**C**) The maximal number of pATM foci per cell among the 10 min and 1h data shown in panels (**A**,**B**) was plotted against the corresponding CTCAE grade values (closed circles). The best data fit was obtained with the linear law: pATMmax (grade) = 41.72 − 6.78 × grade (r^2^ = 0.74) (dotted line). The points corresponding to the *ATM*- (open circles) and the *LIG4*- (open triangles) mutated cell lines are indicated.

As described elsewhere, and as Section 2.3 suggests, the number of RI γH2AX foci scored 10 min after irradiation in the COPERNIC fibroblasts was found to be systematically lower than those of the radioresistant controls [43]. These data do not suggest that fewer DSBs were physically induced but that fewer DSBs were recognized by the ATM phosphorylation of H2AX histone at the DSB sites via NHEJ. The RIANS model is based on the assumption that any delay in the ATM nucleoshuttling is responsible for radiosensitivity and abnormal individual response [42]. IR triggers the monomerization of cytoplasmic pATM dimers. ATM monomers diffuse in the nucleus, and re-associate as dimers at the DSB sites once the DSBs are recognized [42,49]. From our historical data, an exposure to 2 Gy X-rays generally results in the formation of about 40 pATM foci per cell at 10 min post-irradiation in radioresistant fibroblast controls. In the ATM-mutated cells, no pATM foci were observed. The number of pATM foci, therefore, varies from about 40 to 0 [43]. Such a hypothesis was verified by plotting the pATM foci against the CTCAE. As already reported in a published paper with 117 COPERNIC cell lines [43], the 200 COPERNIC cells tested here showed a maximal number of pATM foci at 10 min or else at 1 h after 2 Gy. Hence, the maximal number of pATM foci reached at 10 min or 1 h post-irradiation, pATMmax, reflects the maximal ATM kinase activity in the nucleus [43]. When plotted against the corresponding CTCAE grades, the number of pATM foci per cell assessed 10 min (Figure 6A) or 1 h (Figure 6B) post-irradiation decreased by obeying a linear law whose correlation coefficient varied and appeared low (r^2^ = 0.70 and r^2^ = 0.38, respectively). Interestingly, the number of pATM foci assessed at 1 h post-irradiation in radioresistant controls were found systematically lower than the 10 min data values and, than those of the other COPERNIC cells, suggesting that DSB repair was already efficient at this post-irradiation time (Figure 6A,B). Furthermore, the dispersion of pATM data for both conditions appeared too large to discriminate data in the CTCAE grade 2 to 4 range (Figure 6 A,B). Such findings reflect the differences in the kinetics of the nuclear ATM kinase activity that may exist in human cells. Hence, pATM data assessed either at 10 min or else 1 h post-irradiation cannot predict CTCAE grades reliably. By contrast, by plotting pATMmax with the corresponding CTCAE grade data, a linear function of the grade associated with a good discrimination of CTCAE grades appeared (Figure 6C) (Table 1):

pATMmax (grade) = 41.72 − 6.78 × grade; r^2^ = 0.74 (4)

Interestingly, such a mathematical formula suggests that decreasing every 6.78 arly pATM foci per cell increment leads to a decrease in one additional CTCAE grade (Figure 6C). Such conclusions did not significantly depend on whether the *LIG4*-mutated cell data are integrated or not in the calculations.

It must be stressed that, among the molecular endpoints tested here, the pATMmax provided the best discrimination of the CTCAE grades (Table 2).

**Figure 7:**
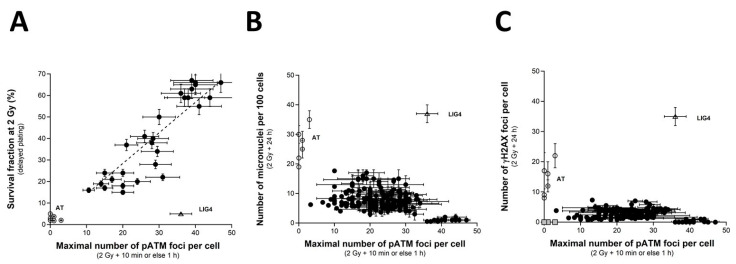
SF2 vs. pATMmax, pATmax vs. MN_24h_ and pATMmax vs. H2AX_24h_ relationships. (**A**) The SF2 data from 36 COPERNIC cell lines were plotted against the corresponding number of γH2AX foci per cell assessed 24 h post-irradiation (closed circles). Each point corresponds to the mean ± standard error of the mean (SEM) of three independent triplicates, at least. The best data fit was obtained with the linear law: SF2 (pATMmax) = 1.422 × pATMmax; r^2^ = 0.87 (dotted line). (**B**) The pATMmax from 200 COPERNIC cells were plotted against the corresponding number of micronuclei assessed 24 h post-irradiation (**B**) or the corresponding number of micronuclei assessed 24 h post-irradiation (**C**) Each point corresponds to the mean ± standard error of the mean (SEM) of three independent triplicates, at least. The grey square indicates all the AT data if considering γH2AX foci in *ATM*-mutated cells as absent. The points corresponding to the *ATM*- (open circles) and the *LIG4*- (open triangles) mutated cell lines are indicated.

When SF2 values are plotted against the corresponding pATMmax values, a linear correlation appeared between the two endpoints (Figure 7A) (Table 1):

SF2 (pATMmax) = 1.422 × pATMmax; r^2^ = 0.87 (5)

Such a linear correlation was found consistent with the fact that both SF2 and pATMmax elicited a linear correlation with CTCAE grades. The value of the coefficient of proportionality will be discussed in the next chapters.

Lastly, by plotting the pATMmax with MN_24h_ or else H2AX_24h_, it appeared that the higher the pATMmax, the lower the MN_24h_, and the lower the H2AX_24h_, in coherence with the existence of a linear or a curvilinear law between these last two endpoints (see above chapters) (Figure 7B,C). Again, these last findings showed that data can be discriminated into three distinct categories: the radioresistant cell lines (corresponding to CTCAE grade 0), the hyper-radiosensitive cell lines (corresponding to CTCAE grade 5), and the cell lines showing an intermediate radiosensitivity (corresponding to CTCAE grades 1–4). The case of the unique *LIG4*-mutated cell lines can be considered as a fourth category.

### 2.5. A Global Inter-Correlations System with the Major Radiosensitivity Endpoints

Altogether, our findings confirm the existence of multiple correlations between the major radiosensitivity endpoints, namely the CTCAE grades, SF2, MN_24h_, H2AX_24h_, and pATMmax. If we define max(SF2), min(SF2), and ΔSF2 as the maximal SF2 reflecting the highest radioresistance, the highest radiosensitivity observed in human fibroblasts, and the difference between max(SF2) and min(SF2), respectively:

Formula (1) suggests that:

(6) $$\mathrm{SF}2\;(\mathrm{grade})=\max(\mathrm{SF}2)-\frac{\Delta SF2}{5}\;\mathrm{grade}$$

Formula (6) suggests a solution of the following differential equation:

(7) $$\frac{dSF2\;\left(grade\right)}{dgrade}=-k0$$

in which:

(8) $$k_{0}=\frac{\Delta SF2}{5}$$

If we define max(MN_24h_) as the maximal MN_24h_ value:

Formula (2) suggests that:

(9) $$SF2\;\left(MN_{24h}\right)=\max\left(SF2\right)e^{-k_{1}MN_{24h}}$$

in which:

k_1_ = ln (max(SF2)/min(SF2))/max(MN_24h_) (10)

Formula (9) is the solution of the following differential equation:

(11) $$\frac{dSF2\;\left(MN_{24h}\right)}{dMN_{24h}}=-k1\;SF2$$

If we define max(H2AX_24h_) as the maximal H2AX_24h_:

Formula (3) suggests that:

(12) $$\mathrm{SF}2\;\left(H2{\mathrm{AX}}_{24h}\right)=\max\left(\mathrm{SF}2\right)e^{-k2\;H2{\mathrm{AX}}_{24h}}$$

in which:

k2 = ln (max(SF2)/min(SF2))/max(H2AX_24h_) (13)

Formula (12) is the solution of the following differential equation:

(14) $$\frac{\mathrm{dSF}2\;\left(H2{\mathrm{AX}}_{24h}\right)}{\mathrm{dH}2{\mathrm{AX}}_{24h}}=-k2\;\mathrm{SF}2$$

If we define max(pATMmax), min(pATMmax), and ΔpATMmax as the highest, the lowest values taken among the pATMmax, and the difference between max(pATMmax) and min(pATMmax), respectively (i.e., corresponding to the radioresistant controls), Formula (3) suggests that:

(15) $$\mathrm{pATMmax}\;(\mathrm{grade})=\max(\mathrm{pATMmax})-\frac{\Delta \mathrm{pATMmax}}{5}\mathrm{grade}$$

Formula (15) suggests the following differential equation:

(16) $$\frac{dpATMmax\;\left(grade\right)}{dgrade}=-k3$$

in which:

(17) $$k_{3}=\frac{\Delta pATMmax}{5}$$

Formula (5) suggests that:

SF2(pATMmax) = k_4_ × pATMmax (18)

in which:

k_4_ = max(SF2)/max(pATMmax) (19)

Formula (18) is the solution of the following differential equation:

(20) $$\frac{\mathrm{dSF}2\left(\mathrm{pATMmax}\right)}{\mathrm{dpATmax}}=k4$$

It is noteworthy that Formulas (6), (9), (12) and (15) provide:

(21) $${\mathrm{MN}}_{24h}\left(\mathrm{grade}\right)=\ln{\left(\frac{1}{-k0\;\mathrm{grade}}\right)}^{1/k1}$$

(22) $$H2{\mathrm{AX}}_{24h}\left(\mathrm{grade}\right)=\ln{\left(\frac{1}{-k0\;\mathrm{grade}}\right)}^{1/k2}$$

(23) $${\mathrm{MN}}_{24h}\left(H2{\mathrm{AX}}_{24h}\right)=\frac{k2}{k1}H2{\mathrm{AX}}_{24h}$$

(24) $$\mathrm{pATMmax}\left({\mathrm{MN}}_{24h}\right)=\frac{\max\left(\mathrm{SF}2\right)}{k4}e^{-k_{1}{\mathrm{MN}}_{24h}}$$

(25) $$\mathrm{pATMmax}\left(H2{\mathrm{AX}}_{24h}\right)=\frac{\max\left(\mathrm{SF}2\right)}{k4}e^{-k_{2}H2{\mathrm{AX}}_{24h}}$$

Interestingly, all of Formula (11) is in agreement with the shape of the data shown in Figure 2B, Figure 4, Figure 5C and Figure 7B,C.

Altogether, these formulas suggest the following global system:

(26) $$\{\begin{array}{c} \frac{\mathrm{dSF}2\left(\mathrm{grade}\right)}{\mathrm{dgrade}}=-k0\\ \frac{\mathrm{dSF}2\left({\mathrm{MN}}_{24h}\right)}{{\mathrm{dMN}}_{24h}}=-k1\;SF2\\ \frac{\mathrm{dSF}2\left(H2{\mathrm{AX}}_{24h}\right)}{\mathrm{dH}2{\mathrm{AX}}_{24h}}=-k2\;SF2\\ \frac{d\left(\mathrm{pATMmax}\right)}{\mathrm{dgrade}}=-k3\\ \frac{\mathrm{dSF}2\left(\mathrm{pATMmax}\right)}{\mathrm{dpATMmax}}=k4\; \end{array}$$

## 3. Discussion

### 3.1. The Prediction of OR, a Clinical, Technological, Economical, and Legal Issue

In RT, the dose delivered to the tumor is often a compromise between two antagonistic goals: to reach the maximal anti-tumor efficiency and to limit the occurrence and the severity of OR in the locoregional tissues [6,7]. Indeed, OR can alter the patient quality of life during the treatment [8] and even up to several years [9,10]. The occurrence of OR during treatment may also threaten the compliance to RT by not exposing the patient to the full prescribed dose. Before the end of RT, ORs can modify the anatomy of the organs surrounding the target (by generating weight loss during a head and neck treatment for example) and thus lead to a modified dosimetry—inasmuch as tumor volume reduction can modify target dosimetry [63]. If new RT techniques, such as intensity-modulated RT (IMRT), stereotactic RT with CyberKnife irradiators, protontherapy, or hadrontherapy, tend to improve the risk–benefit balance with a better dose conformation to the target volume, none of them allow for RT without risk for patients [64,65]. Besides, new techniques are not even always superior to reducing ORs [64,66,67]. Above all, according to recent data, it is also noteworthy that ORs may be underestimated by clinicians [68].

**Figure 8:**
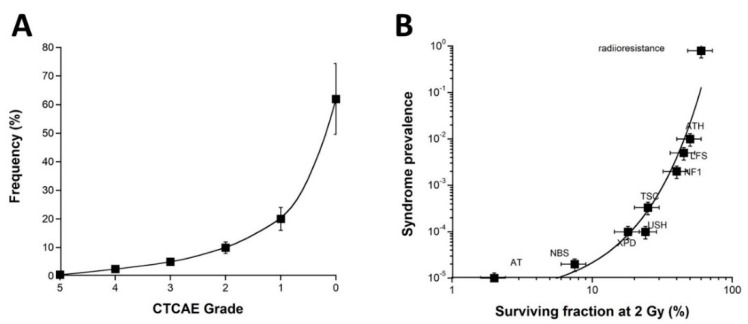
(**A**) Frequency of the OR as a function of the CTCAE grades. These data were established from the COPERNIC collection ([43] and N.F. personal communication). (**A**) A 20% relative error was applied to all the data. The best data fit was obtained with an exponential law. (**B**) Prevalence of the indicated genetic syndromes associated with radiosensitivity as a function of SF2 data [43]. These data have been published in a previous report in another form. Error bars were built on the basis of 20% relative error for SF2 and prevalence, respectively. (AT: ataxia telangiectasia, homozygous mutations of *ATM*; NBS: Nijmegen’s syndrome, homozygous mutations of *NBS1*; XPD, xeroderma pigmentosum D, homozygous mutations of *XPD*; USH, Usher’s syndrome, homozygous mutations of USH; TSC, tuberous sclerosis, heterozygous mutation of *TSC*; NF1, neurofibromatosis type 1, heterozygous mutations of neurofibromin; LF2, Li-Fraumeni’s syndrome, heterozygous mutations of *p53*; ATH, heterozygous mutations of *ATM)*. The best data fit was obtained with an exponential law.

In parallel, it must be stressed that ORs with CTCAE grades 2–5 represent a significant but small subset of post-RT events generally evaluated to about 5 to 20% of patients [3]. Furthermore, the CTCAE grade-distribution of ORs may show a semi-gaussian shape with subsets of CTCAE grades 0, 1, 2, 3, 4, and 5, representing, from previous COPERNIC data and in agreement with the literature [43], about 65, 17, 10, 5, 2.5, and 0.5% of RT-treated patients (with a relative error of about 20% each), respectively. Besides, such estimation strongly depends on whether the CTCAE grade 1 ORs are still considered as tissue reactions without significant clinical consequence or as radiosensitivity reactions. Furthermore, such grade distribution strongly depends on the RT modality and the type of tumor to be treated. The fatal reactions remain very exceptional and are likely due to a genetic predisposition to hyper-radiosensitivity similarly to for *ATM* and *LIG4* mutations [3,15]. Interestingly, the frequency of genetic syndromes as a function of the radiosensitivity level (SF2) reported previously [15] obeys a similar shape to the CTCAE grade distribution of ORs (Figure 8).

Such a statement strengthens again the fact that the clinical, cellular, and biostatistical features of the radiotoxicity observed in RT are similar to those of the radiosensitivity observed with genetic diseases. Another important consequence of such a statement is that the CTCAE grade distribution may introduce some biases in the analysis of the data from clinical studies. Indeed, since severe ORs remain exceptional, the prospective studies may be based on cohorts of RT-treated patients with a majority of radioresistant patients and a minority of radiosensitive ones. Consequently, in this case, the statistical robustness of any predictive assay may be overestimated for the lowest grades and underestimated for the highest ones. Conversely, the retrospective studies can be based on a specific distribution of the cohorts: hence, the best compromise would be to obtain the same number of cases for each CTCAE grade to ensure an OR prediction that would not be dependent on the severity grade of the OR [3]. Further investigations are needed to optimize the methodology and all these statistical constraints.

### 3.2. The Diverse Predictive Assays and Their Associated Endpoints

There is a large body of evidence that ORs are the clinical consequences of RI cell death [3,6,16,17]. The clonogenic survival assay, first developed in 1956 by Puck and Markus, is the current measurement of RI death in vitro [69,70]. In 1981, a quantitative correlation between tumor radio responsiveness (quantified in vivo by using tumor local control as an endpoint) and cellular radiosensitivity (quantified in vitro by using SF2 as an endpoint) was pointed out for the first time, validating the clonogenic cell survival assay as the most reliable predictive assay of anti-tumor efficiency of the RT [24]. The clonogenic cell survival assay has been also applied to a number of non-tumor cellular models, notably lymphoblasts and fibroblasts [26,27,71]; in 1975, Taylor et al. pointed out, for the first time with the clonogenic cell survival assay, the extreme radiosensitivity associated with ataxia telangiectasia (*ATM* mutations) [45]. After this discovery, a considerable number of studies have reported the radiobiological characterization of various genetic syndromes which permits to propose, to date, a complete view of human radiosensitivity [22,52,53,54,72,73,74,75,76]. Conversely, with regard to the radiobiological characterization of cells provided from OR patients, there are a few reports about the link between clonogenic cell survival data and the CTCAE grade. For example, Pouliliou et al. (2015) investigated SF2 in peripheral blood lymphocytes from RT-treated patients. However, the corresponding CTCAE grades of this study were grouped into three categories of grades of early reactions (0, 1, and 2 + 3) [77]. To our knowledge, no report with SF2 has involved the full range of CTCAE grades like our work published in 2016 [43] and this present study. Lastly, it must be stressed that the range of SF2 observed in cells from OR patients is similar to that observed in cells from patients suffering from genetic syndromes, which demonstrates that the two phenomena are similar. Besides, all the young *ATM*- or *LIG4*-mutated patients suffering from hyper-radiosensitivity and treated with RT succumbed by eliciting CTCAE grade 5 ORs [45,60,78,79,80]. To date, the clonogenic cell survival assay as a predictive assay for OR occurrence and severity has been abandoned because it is based on a long procedure of cell culture: (1) the plating efficiency of each cell line tested must be determined with precision by seeding a series of numbers of non-irradiated cells and scoring the resulting colonies that generally appear in 7 to 14 days; (2) in order to avoid the feeder effects, the concentration of cells giving the highest plating efficiency is then deduced; (3) a series of numbers of irradiated cells are seeded and the resulting colonies and fractions of cell survival are calculated. Hence, the clonogenic cell survival protocol requires some weeks, which means its routine clinical application is very complex [70].

**Figure 9:**
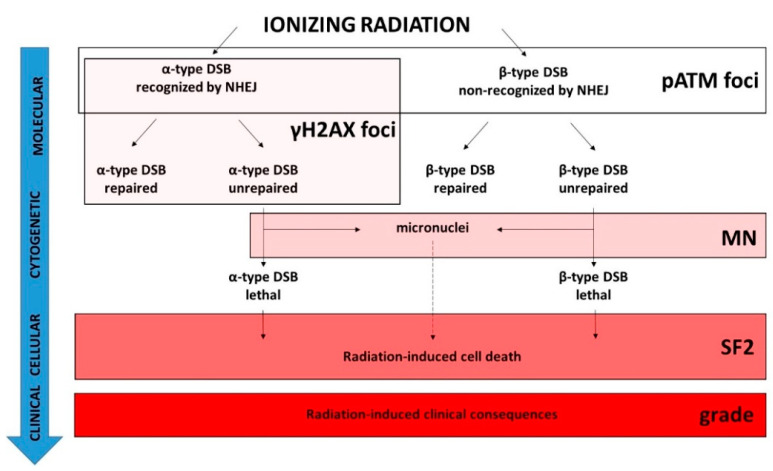
Schematic view of the molecular, cytogenetic, cellular, and clinical consequences of exposure to IR and the validity domain of the major radiosensitivity endpoints. IR induces two types of DSB. The α-type DSBs are recognized by the NHEJ DSB repair pathway while the β-type DSBs are not [49]. For each type of DSB, there are some subsets of unrepaired DSB [49]. Among them, some may be unrepairable and contribute to the lethal effect. Some unrepaired DSBs may also provide micronuclei according to the radiosensitivity status and the capacity of irradiated cells to bypass the G2/M arrest [15]. The pATM foci biomarker detects the DSBs recognized by NHEJ, and the DSBs non-recognized by NHEJ or recognized by another DSB repair pathway can be deduced from the induction rate of DSBs “physically” induced by IR. The γH2AX foci biomarker detects the α-type DSBs only. Both α- and β-type unrepaired DSBs may provide some micronuclei, but the ratio between unrepaired DSBs and micronuclei is not necessarily equal to 1. Some subsets of micronuclei can contribute to the lethal effect. SF2 reflects all the RI cell deaths and therefore reflects the whole cellular response to IR independently of the DSB repair pathways involved. The dashed line indicates that the link is different from a one-to-one correlation.

The clonogenic cell survival accounts for the whole RI cell death and is not specific to a particular cell death pathway (Figure 9). The major RI cell deaths are mitotic death, senescence, and apoptosis [3]. Mitotic death is predominant in cells that can proliferate, whether that be fibroblasts or lymphocytes. Micronuclei are one of the current endpoints reflecting mitotic death and they have been observed when NHEJ or any other DSB repair pathways (like homologous recombination) were impaired, suggesting that micronuclei do not occur only when NHEJ is faulty [3,51] (Figure 9). Furthermore, the occurrence of a micronucleus requires not only unrepaired DNA breaks but, overall, a bypass of G2/M arrest. However, radiosensitivity may be independent of the G2/M arrest status [15]. For example, the ratio between unrepaired breaks and micronuclei may drastically differ between cells from aging syndromes (generally arrested in G0/G1) and cells from cancer syndromes (generally impaired in G2/M arrest) [15]. Together with clonogenic assays, cytogenetic assays, similarly to the micronuclei assay, were initially based on the staining techniques like Giemsa and require metaphases and therefore a significant period of time to reach new cell cycle phases [28]. Hence, the most current protocol for scoring micronuclei is based on the cytokinesis block with cytochalasin B that accelerates the passage in the G2/M phase. However, this step may introduce bias by mixing the capacity of cells to pass G2/M (and therefore by artificially increasing the micronuclei production rate) with the dose-dependent ratio between micronuclei and unrepaired chromosome breaks [28,81,82,83]. To overcome such bias, the micronuclei assay protocol applied in the present study did not involve any artificial block cells in G2/M but followed the same protocol as that applied in γH2AX and pATM immunofluorescence to better facilitate data inter-comparisons (see materials and methods). A high number of unrepaired chromosome breaks (and therefore micronuclei) has been shown to characterize many radiosensitive genetic syndromes [34,83,84,85]. Conversely, the great majority of studies failed to confirm the applicability of the micronuclei assay to predict OR for RT-treated patients [83]. Furthermore, to our knowledge, no report has investigated the relationships between yields of micronuclei and a large range of CTCAE grades. This short review is consistent with the fact that the micronuclei assay is unable to discriminate the CTCAE grades 1 to 4 ORs reliably. The fact that Cornforth and Bedford demonstrated that one unrepaired chromosome break corresponds to one lethal event for non-transformed human fibroblasts does not contradict the general tendency observed in the present study (*the higher the number of micronuclei*, *the higher the radiosensitivity, the more severe the OR*) but illustrates well that the ratio between unrepaired DSB and micronuclei may differ among the human cell lines according to their radiosensitivity status and their capacity to bypass the G2/M arrest [15,34].

In coherence with the causal links between SF2, micronuclei, and unrepaired DSBs, there is a plethora of studies aiming to characterize radiosensitivity with DSB repair assays [3,15,36,37]. In the 1990s, radiobiologists focused on the yield of unrepaired DSBs as a potential radiosensitivity predictor by using notably pulsed-field gel electrophoresis (PFGE), comet, and immunofluorescence techniques [12,36]. The major advantage of DSB repair assays based on the assessment of RI DNA fragmentation like PFGE, elution, or sucrose sedimentation is that they provide data independent of any specific DSB repair pathway. Conversely, their major inconvenience is that they require very high doses (often non-biologically relevant) to allow the DNA breaks to be detectable, which raises the question of the dose-dependence of the DSB repair rate when assessed by these techniques [3]. By contrast, the γH2AX immunofluorescence assay requires the same dose range as those applied in RT, clonogenic cell survival, and micronuclei assays. However, does each nuclear γH2AX foci correspond to one DSB, whatever the cell lines, their radiosensitivity status, and the irradiation conditions? As evoked in Section 2.3, the formation of γH2AX foci is ATM-dependent and represents a major early step of NHEJ. The number of γH2AX foci assessed per Gy early after irradiation is similar to the number of DSBs induced per Gy by using PFGE [38,41,55,56,57,58]. However, such an observation has been performed with the radioresistant quiescent human cells and with the *LIG4*-mutated cell lines that show normal ATM kinase activity [42,43]. Conversely, in radiosensitive cells that show impaired ATM kinase activity, the number of early γH2AX foci was shown to be lower than the number of DSBs “physically” induced by IR, suggesting an impairment in DSB recognition by NHEJ in these cells [42,43]. Hence, the γH2AX foci represent a limited subset of all the RI DSBs: the DSBs recognized by NHEJ only. Consequently, the residual (or persistent) γH2AX foci observed 24 h post-irradiation do not necessarily represent all the DSBs that contribute to the RI lethal effect (Figure 9). The RIANS model has integrated two types of RI and lethal DSBs [49]: (1) the α-type DSBs, recognized by the ATM monomers in the nucleus (therefore by NHEJ) early after irradiation (presence of γH2AX foci). Some of them may remain unrepairable and contribute to the RI lethal effect (persistent γH2AX foci). Their number was demonstrated to be proportional to the dose [49]; (2) the β-type DSB, not recognized by the ATM monomers in the nucleus (therefore not managed by NHEJ) because of a delay or an absence of the RIANS. Some of them may remain unrepairable and contribute to the RI lethal effect. However, these DSBs are not visible by using γH2AX immunofluorescence. Their number was demonstrated to be proportional to the square of the dose [49]. As a result, the number of all these lethal DSBs is the same as the linear-quadratic expression found in the LQ model [49] (Figure 9). Besides, such a definition of α- and β-type DSBs is also consistent with the radiobiological features of the unique *LIG4*-mutated 180BR cell line.

How does one explain the pATM data and their prediction power? As evoked in Section 2.3 and Section 2.4, the fact that less early γH2AX foci were observed in the radiosensitive COPERNIC cells does not suggest that fewer DSBs are induced “physically” in these cells but, rather than fewer DSBs are recognized by the ATM-dependent phosphorylation of H2AX, consistently with lower nuclear ATM kinase activity, caused by a delay in the RIANS [43]. In a previous report, with 117 COPERNIC cell lines, the correlation between pATMmax, and the CTCAE grade was found to be significant (concordance coefficient: *p* = 0.86) [43]. By adding here 83 additional COPERNIC fibroblasts, the statistical robustness of the correlation between pATMmax and the CTCAE grade was confirmed (Figure 6). To our knowledge, there is no equivalent of such correlation in the literature with the complete range of CTCAE grades. Logically, by considering a binary approach, i.e., by gathering OR of grades 0, 1, and 2 in one category and grades 3, 4, and 5 in another category, the superiority of pATMmax and SF2 in the prediction of ORs was also found to be enhanced, in agreement with previous reports [43,47,48] (Table 3). Since our group particularly focuses on the RIANS model, there is no equivalent of SF2-pATMmax, MN_24h_-pATMmax, and γH2AX_24h_-pATMmax correlation in the literature either. Our findings strongly suggest that, with SF2, pATMmax appears to be the most powerful predictor of the CTCAE grade ORs.

The conclusion that both SF2 and pATMmax appear to be the best predictors of ORs has been reached but not simply because a linear function has been found between these endpoints and the CTCAE grades. Some correlation coefficients may be higher with other mathematical laws and other endpoints. The high prediction power of the pATMmax endpoint is based on the fact that the pATM foci account for all the DSBs managed by NHEJ and that the number of the DSBs managed by other DSB repair pathways, if any, can be easily estimated from the well-documented DSB induction rate of about 40 DSBs per Gy per human untransformed fibroblast [42]. Hence, by integrating all the RI DSBs that may potentially contribute to the lethal effect, independently of any DSB repair pathway involved or impaired, both SF2 and pATMmax provide a more exact view of the radiobiological response of human cells. The other endpoints (micronuclei and γH2AX foci) reflect only a limited subset of the RI DSBs (Figure 9). In addition to this explanation, it must be also stressed that both SF2 and pATMmax vary in ranges ([3–62%] and [0–42 pATM foci], respectively) larger than those of the other endpoints tested. Hence, the different levels of cellular and molecular response to IR can be better discriminated with SF2 and pATMmax [3,42].

### 3.3. The Detection of Radiosensitivity, a Unique Multiparametric System?

The mathematical approach applied in this study consisted in:

- considering that some causal or partially causal link documented by the literature exists between each of the endpoints tested.
- the nature of such links can be reflected by a specific mathematical link between each of the endpoints tested.
- the data-fitting analysis was conducted with some current mathematical laws (linear, curvilinear, exponential, and power functions). Hence, some other (but more complex) mathematical laws can be tested in further investigations.
- we have considered that the best data fit was the solution of a differential equation linking two endpoints.
- the k-coefficient, the type, and the order of each differential equation found were hypothesized to reflect the complexity of the link between the two endpoints considered.

The differential equations described in the last sections of the Results chapter may suggest Michaelis–Menten equations. However, the CTCAE grades, the cell survival (SF2), the number of micronuclei, and the number of γH2AX and pATM foci do not represent the same scale (cell, chromosome, DNA, and proteins, respectively) and these endpoints cannot be considered as interplaying substrates: consequently, the theory of enzymatic kinetics is not applicable here.

**Figure 10:**
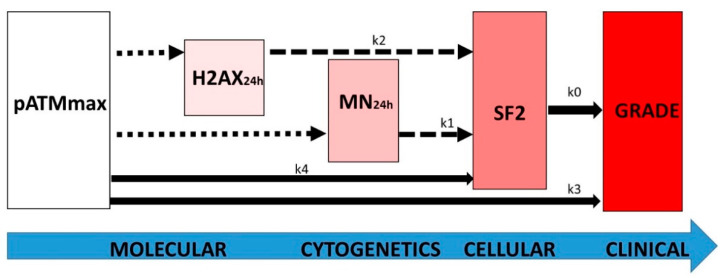
Schematic view of the link between the major radiosensitivity endpoints with the k coefficients of the differential equations described at the end of the Results chapter. The dotted, dashed, and full-line arrows represent complex, exponential, and linear laws, respectively.

Conversely, the biological features and specificities of each endpoint tested (summarized in Figure 9) and their mathematical constraints may lead to the following interpretations that illustrate the direct (linear) and indirect or incomplete (non-linear) links between the endpoints tested (Figure 10):

- the k0 coefficient represents the direct link between the RI cellular death (SF2) and the clinical OR (grade). It illustrates that any clonogenic cell death corresponds to an RI tissue event. The mathematical constraints are simple: the k0 coefficient only depends on the maximal range of the scale (i.e., six grades and therefore five inter-grade intervals for CTCAE) and the range of SF2 (i.e., ΔSF2) (see Formula (8). Quantitatively, decreasing every 11.72% cell survival increment leads to a decrease in one CTCAE grade.
- the k3 coefficient represents the direct link between the maximal nuclear RI ATM kinase activity (pATMmax) and the clinical OR (grade). It illustrates the fact that the pATM foci lacking (due to the non-recognition of DSBs by NHEJ or by another DSB repair pathway) directly represent a subset proportional to the number of lethal DSBs responsible for an RI tissue event. As for k0, the k3 coefficient only depends on the maximal range of the scale (i.e., six grades and therefore five inter-grade intervals for CTCAE) and the range of pATMmax (i.e., ΔpATMmax) (see Formula (17). Quantitatively, decreasing every 6.78 pATM foci per cell increment leads to a decrease of one CTCAE grade.
- the k4 coefficient supports that the maximal nuclear ATM kinase activity (pATMmax) directly conditions cell survival: the higher the pATMmax, the higher the SF2. As specified above, pATMmax reflects the maximal nuclear RI ATM kinase activity. By combining Formulas (6) and (15), the k4 coefficient appears to directly depend on k0, k3, max(SF2), and max(pATMmax). Quantitatively, decreasing every 6.78 early pATM foci per cell increment leads to a decrease of 11.72% cell survival.
- the k2 coefficient represents the number of DSBs that contribute to the RI lethal event among the unrepaired DSBs reflected by persistent γH2AX foci (i.e., recognized by NHEJ) (Figure 9). Since k2 = 0.21, our findings suggest that about one event per five unrepaired DSBs recognized by NHEJ may be lethal. Such interpretation leads to the notion of the tolerance of DSBs: some unrepaired DSBs may be not lethal, which is in agreement with previous reports and the current observation that some cells can elicit a significant number of spontaneous DSBs without impacting on their capacity of repair [30,43]. It is noteworthy that Formula (22), deduced from the other formulas, fits well when the H2AX_24h_ data are plotted against the CTCAE grades (Figure 4).
- the k1 coefficient represents the number of micronuclei per 100 cells that contribute to the RI lethal event among all the micronuclei detected. Since k1 = 0.107, our findings suggest that about 1 per about 10 micronuclei observed per 100 cells is lethal. As for H2AX_24h_, such interpretation also leads to the notion that some micronuclei may not contribute to the RI lethal event, maybe by enhancing the transformation (misrepair) of the cells [30,43]. It is noteworthy that Formula (21), deduced from the other formulas, fits well when the MN_24h_ data are plotted against the CTCAE grades (Figure 3). Interestingly, the link between MN_24h_ and H2AX_24h_, illustrated by Formula (23), suggests that there is a constant subset of unrepaired DSBs reflected by γH2AX foci that leads to the formation of micronuclei. According to Formula (23), this rate suggests that all the unrepaired DSBs recognized by NHEJ do not lead to the formation of micronuclei.

Interestingly, the resulting equations system linking the major radiosensitivity endpoints described by Formula (26) may be also relevant for all mammalian cells, especially for rodent models. Indeed, the k coefficients linking the SF2, MN_24h_, H2AX_24h_, and pATMmax endpoints were shown to depend on their maximal and minimal values. It must be stressed that the most radioresistant and the most radiosensitive rodent cellular models show similar bounded values as those observed in humans. For example, SF2 is generally limited to 1 to 80% in mammalians. This is also the case for the yields of residual micronuclei and unrepaired DSB yields [40].

## 4. Materials and Methods

### 4.1. Fibroblast Cell Lines

All of the experiments were performed with untransformed skin fibroblast cells in the plateau phase of growth under standard culture conditions described elsewhere [43,54]. Skin biopsies sampling was performed in unirradiated areas (generally under the forearm) after local anesthesia, similarly to standardized dermatologic punch. All the anonymous patients were informed and gave signed consent according to the ethics recommendations. Clinical data on tumor characteristics and therapy regimens were extracted from the medical records. The OR severity was graded by two independent clinicians according to the Common Terminology Criteria for Adverse Events (CTCAE) version 4.03. Only OR patients with consensual clinical grading were included in this study. Both early and late reactions were considered. Cancer patients suffered from breast, prostate, nose ear throat, lymphoma, nervous system (central and peripheral), lung, anal canal, pediatrics, cervix, sarcoma, skin, testis, bone, rectum, and esophagus cancer. In order to avoid bias, no tumor type subset represented more than 40% of the collection. There was no correlation between the CTCAE grade and age, sex, regimen, or total cumulated dose of the treatment. All the sampling procedures were done in the frame of the experimental protocol of the “COPERNIC” collection, approved by the national ethical committee in agreement with the current national regulations about the clinical studies. The resulting fibroblast cell lines were declared under the numbers DC2008-585, DC2011-1437, and DC2021-3957 to the Ministry of Research. This study involved 200 COPERNIC fibroblast cell lines, including:

- A total of 117 COPERNIC cell lines already described in a published report. This subset is composed of 12 radioresistant, 4 ATM-mutated, and 1 LIG4-mutated gifted cell lines and the 100 first registered cell lines of the COPERNIC collection derived from RT-treated patients who showed grades 1–4 CTCAE ORs. The Radiobiological Database of this subset is protected under the reference IDDN.FR.001.510017.000.D.P.2014.000.10300
- A total of 82 additional COPERNIC cell lines. This subset is composed of 2 additional radioresistant, 2 ATM-mutated cell lines, and 78 cell lines of the COPERNIC collection derived from RT-treated patients who showed grades 1–4 CTCAE ORs that were chosen randomly in a subset of 150 available ones. The protection procedure of the radiobiological database of this subset is in progress.

### 4.2. X-rays Irradiation

Irradiations were performed with a 6 MeV X-ray medical irradiator (SL 15 Philips) (dose-rate: 6 Gy·min^−1^) at the anti-cancer Centre Léon-Bérard (Lyon, France) [43,86]. In all the experiments, a dose of 2 Gy was chosen because it simulates a current dose per session in a standard radiotherapy. The dosimetry was certified by radiophysicists of the Centre Léon-Bérard.

### 4.3. Clonogenic Cell Survival

The intrinsic cellular radiosensitivity was quantified from clonogenic cell survival data obtained from standard delayed plating procedures that were described elsewhere [24]. Cells in the plateau phase of growth were irradiated at the indicated doses, incubated for 24 h at 37 °C, harvested, counted using hemocytometer, and then diluted to a pre-defined number of cells to be seeded in Petri dishes. After 15 days at 37 °C in a CO_2_ incubator, the cells were and stained in crystal violet. Only the colonies with more than 50 cells were scored. The survival data were fitted to the linear-quadratic (LQ) model that describes the cell survival S as a function of dose D, as follows: $S=e^{-\left(\alpha D+\beta D^{2}\right)}$, in which α and β are adjustable parameters to be determined. The intrinsic radiosensitivity was quantified by calculating the surviving fraction at 2 Gy (SF2) [49].

### 4.4. Immunofluorescence

The immunofluorescence protocol and nuclear protein foci scoring was described elsewhere [43,54]. Anti-*γH2AX^ser139^* antibody (#05-636; Merck Millipore, Burlington, VT, USA) was used at 1:800. The monoclonal anti-mouse anti-*pATM^ser1981^* (#05-740) from Merck Millipore was used at 1:100. Incubations with anti-mouse fluorescein (FITC) and rhodamine (TRITC) secondary antibodies were performed at 1:100 at 37 °C for 20 min. By following the same procedure, micronuclei were scored on the same slides by using 4′,6′Diamidino-2-Phényl-indole (DAPI)-counter staining. Foci and micronuclei were scored by eye with an Olympus BX51 fluorescence microscope. For each of the three independent experiments, 100 nuclei were analyzed. The patented procedures of foci scoring have been detailed elsewhere [26]. It is noteworthy that post-irradiation times indicated in the text represent an equal period of time of the incubation of cells at 37 °C without any genotoxic stress (i.e., under standard culture conditions). Such a period is currently considered to be a time for repair [43,54].

### 4.5. Statistical Analysis

Statistical analysis was performed by using Kaleidagraph v4 (Synergy Software, Reading, PA, USA), Graphpad Prism (San Diego, CA, USA) and MATLAB R2020B (MathWork, Natick, MA, USA). Since each experiment is the result of three independent replicates, the mean is given with the standard error of the mean (SEM) of the three independent experiments. The discrimination power of each molecular endpoints was performed with the one-way ANOVA test.

The mathematical analysis of correlations between the different endpoints was based on the following procedure: (1) The data fitting analysis was attempted with the current mathematical laws (linear, curvilinear, exponential, and power functions). The adjustable parameters and the quality of fit were calculated systematically. If the data fitting is not acceptable, the link between the two endpoints is considered as “complex” and is thereafter deduced from the other links established (crossed resolution). (2) The differential equation whose solution is provided by the best data fit is established. (3) The k-coefficient and all the bounded values were deduced numerically.

## 5. Conclusions

By analyzing radiobiological data from 200 skin fibroblast cell lines from RT-treated patients showing a large spectrum of OR severity grades obtained with the major assays predicting radiosensitivity, it appears that SF2 and the maximal number of early pATM foci are the best predictors of all the CTCAE grades while the number of residual micronuclei and γH2AX foci do not predict well the intermediate grades. These findings are consistent with the fact that the clonogenic cell survival assay account for all the dead cells, independently of the cell death pathway. Similarly, the pATM immunofluorescence permits the quantification of DSBs unrecognized by the predominant NHEJ pathway, which directly impact on the RI cell death. Conversely, the residual micronuclei and the γH2AX foci correspond to a limited subset of lethal DSB. Gathered all in a differential equations system, these major radiosensitivity endpoints are mathematically linked in a single mechanistic model of individual response to radiation in which the ATM kinase plays a major role. Further investigations are needed to better exploit this system in the prediction of the deleterious effects of any exposure of mammalian cells to IR and, more generally, to any DSB-inducer agent.

## Figures and Tables

**Table 1:** Fitting parameters for the relationships between the radiosensitivity endpoints.

Endpoints Concerned	Fitting Function	Adjusted a1 Value *	Adjusted a2 Value *	Adjusted r^2^	SSE **	RMS ***
SF2 = f(grade)	f(x) = a*x + a2	−11.72 (−12.3, −11.15)	61.55 (59.77, 63.32)	0.9805	329.9	3.162
SF2 = f(MN_24h_)	f(x) = a*exp(b*x)	68.55 (62.39, 74.72)	−0.1142 (−0.1363, −0.09208)	0.8839	1966	7.719
SF2 = f(H2AX_24h_)	f(x) = a*exp(b*x)	62.56 (57.25, 67.87)	−0.2168 (−0.261, −0.1726)	0.8685	2228	8.217
pATMmax = f(grade)	f(x) = a1*x + a2	−6.786 (−7.356, −6.215)	41.72 (39.99, 43.45)	0.7399	3912	4.514
SF2 = f(pATMmax)	f(x) = a1*x	1.422 (1.322, 1.523)	NA	0.8794	1971	7.848

* with 95% confidence bounds; ** SSE: sum squared error; *** RMSE: root mean square error; NA: non-applicable. See also Materials and Methods.

**Table 2:** Discrimination power of the major molecular radiosensitivity endpoints *.

Compared CTCAE Grades	*p* Values for MN_24h_	*p* Values for H2AX_24h_	*p* Values for pATMmax
2,3 and 4	0.045	0.0115	8.55 × 10^−30^
2 and 3	0.140	0.018	4.15 × 10^−6^
3 and 4	0.055	0.017	6.51 × 10^−20^
2 and 4	0.030	0.81	2.09 × 10^−24^

* quantified by one-way ANOVA test. See also Section 4.

**Table 3:** Discrimination power of the major molecular radiosensitivity endpoints in a binary approach *.

*p* Values for SF2	*p* Values for MN_24h_	*p* Values for H2AX_24h_	*p* Values for pATMmax
1.90 × 10^−12^	1.52 × 10^−6^	1.51 × 10^−4^	9.91 × 10^−20^

* Patients were divided into two groups, radioresistant (CTCAE scores of 0, 1, and 2) and radiosensitive (CTCAE scores of 3, 4, and 5). A One-Way ANOVA test was performed to assess the discrimination power of each molecular endpoint.

## Data Availability

All the data can be provided on reasonable request.
